# The outcome at follow-up after inpatient eating disorder treatment: a naturalistic study

**DOI:** 10.1186/s40337-020-00349-6

**Published:** 2020-12-02

**Authors:** Marit Danielsen, Sigrid Bjørnelv, Siri Weider, Tor Åge Myklebust, Henrik Lundh, Øyvind Rø

**Affiliations:** 1 Eating Disorder Unit, Department of Psychiatry, Levanger Hospital, Hospital Trust Nord-Trøndelag, NO-7600 Levanger, Norway; 2 Department of Mental Health, Faculty of Medicine and Health Sciences, Norwegian University of Science and Technology (NTNU), Trondheim, Norway; 3 Department of Psychology, Norwegian University of Science and Technology (NTNU), Trondheim, Norway; 4 Department of Research and Innovation, Møre og Romsdal Hospital Trust, Ålesund, Norway; 5 Regional Eating Disorder Service, Division of Mental Health and Addiction, Oslo University Hospital, Oslo, Norway; 6 Division of Mental Health and Addiction, Institute of Clinical Medicine, University of Oslo, Oslo, Norway

**Keywords:** Adults, Eating disorders, Follow-up, Inpatient treatment, Remission, Treatment outcome, Outcome predictors

## Abstract

**Background:**

Patients with eating disorders may experience a severe and enduring course of illness. Treatment outcome for patients provided with inpatient treatment is reported as poor. Research to date has not provided consistent results for predictors of treatment outcome. The aims of the study were to investigate rates of remission at follow-up after inpatient treatment, symptom change from admission to follow-up, and predictors of treatment outcome.

**Methods:**

The follow-up sample consisted of 150 female adult former patients (69.4% of all eligible female patients) with eating disorders. Mean age at admission was 21.7 (SD = 4.9) years. Diagnostic distribution: 66% (*n* = 99) anorexia nervosa, 21.3% (*n* = 32) bulimia nervosa and 12.7% (*n* = 19) other specified feeding or eating disorder, including binge eating. Data were collected at admission, discharge and follow-up (mean 2.7 (SD = 1.9) years). Definition of remission was based on the EDE-Q Global score, body mass index and binge/purge behavior. Paired T-tests were performed to investigate change over time. Univariate and multivariate logistic regressions were estimated to investigate predictors of remission.

**Results:**

At follow-up, 35.2% of the participants were classified as in remission. Significant symptom reduction (in all patients) (*p* <  0.001) and significant increase in body mass index (BMI) (in underweight participants at admission) (*p* <  0.001) was found. Increased BMI (*p* <  0.05), the level of core eating disorder symptoms at admission (*p* <  0.01) and reduced core eating disorder symptoms (*p* <  0.01) during inpatient treatment were found significant predictors of outcome in the multivariate model.

**Conclusions:**

All participants had an eating disorder requiring inpatient treatment. Approximately one-third of all participants could be classified as in remission at follow-up. However, most participants experienced significant symptom improvement during inpatient treatment and the improvements were sustained at follow-up. Increased probability of remission at follow-up was indicated by lower core ED symptoms at admission for all patients, raised BMI during admission for patients with AN, and reduced core ED symptoms during inpatient treatment for all patients. This finding contributes important information and highlights the importance of targeting these core symptoms in transdiagnostic treatment programs.

## Plain English summary

The outcome of specialist inpatient treatment was investigated in Norway during a follow-up study of 150 adult female patients with eating disorders. All patients had an eating disorder that required inpatient treatment at admission to the same specialist eating disorder inpatient unit: 99 were diagnosed with anorexia nervosa, 32 had bulimia nervosa and 19 had an unspecified eating disorder. The sample comprised 69.4% of all eligible female patients. The mean age of the participating patients at admission was 21.7 years. The average duration of their illness was reported as 5.7 years. The investigated data were collected at admission, at discharge and at follow-up, which was completed on average 2.7 years after discharge. Approximately one-third of the patients were classified as in remission when discharged from treatment. The results indicated considerable symptom improvement during inpatient treatment, which was maintained at follow-up. Increased probability of remission at follow-up was indicated by lower core ED symptoms at admission for all patients, raised BMI during admission for patients with AN, and reduced core ED symptoms during inpatient treatment for all patients. This finding contributes important information and highlights the importance of targeting these core symptoms in transdiagnostic treatment programs.

## Background

In order to find more satisfactory treatment results for patients with eating disorders (EDs) and to tailor treatment according to the needs of individual patients it is important that we continue to investigate treatment outcomes. Although most ED patients will benefit from outpatient treatment, we know that a percentage of the population will need inpatient care. Both the American Psychiatric Association (APA) guidelines [1], and the National Institute for Health and Care Excellence (NICE) guidelines [2], recommend inpatient treatment as one treatment option in cases of severe illness (psychological and medical symptoms), enduring illness, and unsuccessful outcome of treatment. Symptom improvement during inpatient treatment of adult patients with an ED enrolled in different treatment programs has been reported [3–11], and findings showed that improvement was mainly sustained at follow-up [3, 6, 8–11]. However, outcome after inpatient treatment is reported as poor [12]. Reports of remission and recovery vary across studies, and the results might have been influenced by lack of standardized definitions and differences in samples, for example regarding the patients’ severity of illness, age, and diagnosis, and follow-up period and statistical analyses [13–17]. For example, remission rates showed variation in the range of 24–55% in one ED sample, depending on how remission was defined [18].

In general, there have been inconsistent research results regarding predictors of treatment outcome in ED samples [17], and results regarding inpatient adult samples are limited. However, some factors of importance for a positive outcome at follow-up have been reported, such as higher body mass index (BMI) at admission (for AN patients) [8, 19], higher BMI at discharge (for AN patients) [11, 19], lower age at admission [8, 9], length of inpatient stay [20], and duration of illness and follow-up period [8, 9].

The findings reported in the literature underline the need for more effective inpatient treatment of the most severely ill ED patients [12]. To achieve this goal, both increased knowledge of facilitating and impeding factors in the course of the illness and replication of research finding are essential [17]. To contribute to existing knowledge of the course of EDs and predictors of remission among adult inpatients, we investigated a transdiagnostic adult ED population admitted for specialist inpatient treatment. The aims of the naturalistic follow-up study were:

1. To report the rate of remission at follow-up
2. To investigate predictors of remission at follow-up.

According to previous prognostic findings reported in the literature, we hypothesize that low age, short duration of illness, and increased BMI (for AN patients), ED symptomatology, and general psychopathology during admission predict remission at follow-up.

## Methods

### Participants and procedure

**Fig. 1:**
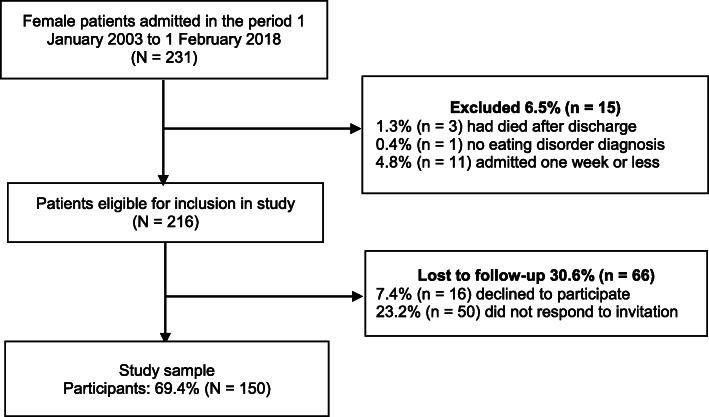
Flowchart of the study sample

Eligible participants for the follow-up study were female patients who had been admitted voluntarily and treated in a specialist inpatient unit for ED in the period 1 January 2003 to 1 February 2018. Other than medically unstable patients, the unit did not have exclusion criteria such as low BMI or comorbid disorders for acceptance for treatment. Between 1 January 2003 and 1 February 2018, 231 female patients were admitted to the unit. In addition, 12 male patients were admitted, of which 10 fulfilled the criteria for inclusion in the study and only 4 agreed to participate. Due to the low number of males, we chose to include only females. At the time of the study, the indications for admission for inpatient treatment were symptom severity and lack of satisfactory improvement following earlier treatment. All patients were admitted to an introductory week. Those who were admitted to the unit for more than 1 week in the defined period, fulfilled the criteria for a diagnosis of an ED, and who had signed the treatment agreement form were invited to participate in the study. The investigated sample consisted of 150 female former adult ED patients. The flowchart for the study sample is shown in Fig. 1 and the baseline characteristics of the participants are listed in Table 1. The average duration of illness (self-reported) was 5.7 years (standard deviation (SD) = 4.6, range 1–28 years); 49% of the sample reported a duration of illness of 5 years or more.

**Table 1:** Baseline characteristics of the whole ED sample and in each diagnostic group

Values	Whole sample *N* = 150 Mean (SD)	AN *n* = 99 Mean (SD)	BN *n* = 32 Mean (SD)	OSFED *n* = 19 Mean (SD)	*F*-value *p*-value	Bonferroni post hoc
Age (years) Range	21.7 (4.9) 16.0–46.7	21.1 (4.4) 16.0–6.5	22.0 (3.0) 17.4–29.3	24.7 (8.3) 17.1–46.7	4.52*	AN vs OSFED*
BMI Mean (SD) Range	17.9 (4.3) 11.7–41.7	15.7 (1.6) 11.7–18.5	21.3 (2.6) 17.6–30.2	23.9 (6.5) 18.7–41.7	94.85***	AN vs OSFED*** & BN vs OSFED**
Duration of illness (years) Range	5.7 (4.6) 1–28	4.9 (3.8) 1–20	5.9 (2.6) 2–10	9.7 (8.0) 2–28	9.73***	AN vs OSFED*** & BN vs OSFED**
	n (%)	n (%)	n (%)	n (%)		
**Main occupation**						
Study	94 (62.7)	64 (64.6)	20 (62.5)	10 (52.6)		
Job	334 (22.7)	20 (20.2)	11 (34.4)	3 (15.8)		
Other	22 (14.7)	15 (15.2)	1 (3.1)	6 (31.6)		
**Ability to study/work**						
Able to perform main occupation	35 (23.3)	26 (26.7)	5 (15.6)	4 (21.1)		
Full-time sick leave	55 (36.7)	38 (38.4)	12 (37.5)	5 (26.3)		
Partial sick leave	34 (22.7)	23 (23.2)	7 (21.9)	4 (21.1)		
On benefits ª	22 (14.7)	10 (10.1)	7 (21.9)	5 (26.3)		
Other	4 (2.6)	2 (2.0)	1 (3.1)	1 (5.3)		
Any depression diagnosis (DSM-5)	73 (48.7)	42 (42.4)	18 (56.3)	13 (68.4)		
Any anxiety diagnosis (DSM-5)	19 (12.7)	15 (15.2)	2 (6.3)	2 (10.5)		

*Notes:* One-way analyses of variance (ANOVA) were performed between diagnostic groups (age, BMI and duration of illness), df (2, 147); Bonferroni post hoc test; significant relationships reported; duration of illness self-reported

Due to low numbers, three participants with a binge eating disorder diagnosis were included in the OSFED group

*Abbreviations: BMI* body mass index, *AN* anorexia nervosa, *BN* bulimia nervosa, *OSFED* other specified feeding or eating disorder, *DSM-5* The Diagnostic and Statistical Manual of Mental Disorders

*P* value: * *p* < .05; ** *p* < .01; *** *p* < .001

ª Norway has a national insurance scheme. If loss of income and inability to work are caused by health impairment, Norwegian citizens are offered universal sickness and disability benefits to compensate for the loss

Of the eligible patients, 30.6% were lost to follow-up. There were no significant differences between the participants and the non-participants regarding age at admission (*p* = 0.45) (the whole sample) or BMI at admission (*p* = 0.24) (AN).

The average duration of inpatient stay for the study sample was 140.5 days (SD = 68.7, range 10–340 days). The longest duration was found among anorexia nervosa (AN) patients (mean = 156 days (SD = 74)). The data were collected at admission, discharge and follow-up after the first admission. After discharge, the average follow-up period was 2.7 years (SD = 1.9) for the whole sample. The study was approved by the Regional Committee for Medical and Health Ethics for Central Norway (REC Central) (Project number: 2009/1864). Due to ethical approval being given in 2009, the earliest admitted patients had a prolonged follow-up period. A total of 74% (*n* = 111) had a follow-up period of between one and 5 years. The remaining 39 patients had a follow-up period ≤1 year (*n* = 14) or > 5 years (*n* = 25). At follow-up, the participants completed self-report questionnaires, background information forms and consent forms, and provided self-reported information about weight. The participants’ written informed consent forms were provided in accordance with the Declaration of Helsinki. No compensation was given for participation in the study.

### Assessment

Assessment of the patients at admission to the unit was performed by licensed psychologists and psychiatrists. Furthermore, in team meetings, which were attended by two or more specialists, all diagnoses were discussed until consensus was reached. The diagnoses were based on the unit’s clinical interviews and in accordance with criteria in the fourth edition of the *Diagnostic and Statistical Manual of Mental Disorders* (DSM-IV) [21], which were converted by experienced clinicians (one psychiatrist and one clinical psychologist) to the criteria in the fifth edition, DSM-5 [22]. The clinicians went through the patients` medical records independently and focused their searches on reports of diagnostic evaluations and descriptions of ED criteria and symptoms. In cases when the clinicians disagreed upon the correct diagnosis, medical records were checked again and the diagnoses were discussed until consensus was reached.

Clinical diagnostic evaluation of comorbid disorders of depression and anxiety was classified according to the diagnostic criteria (see Table 1).

The diagnostic distribution of ED at admission was 66% (*n* = 99), anorexia nervosa (AN) 21.3% (*n* = 32) bulimia nervosa (BN), 2% (n = 3) binge eating disorder (BED), and 10.7% (*n* = 16) other specified feeding or eating disorder (OSFED; patients with subthreshold AN (*n* = 8) subthreshold BN (n = 1)). Due to the low number of BED patients, their data were analyzed together with data for patients in the OSFED group. A BMI of 18.5 was used as a threshold value for AN. Among the AN patients, 75.8% (*n* = 75) were restrictive subtype (AN-R), and 24.2% (*n* = 24) bulimic subtype (AN-B). One-way analysis of variance (ANOVA) showed that the OSFED patients were on average significantly older than other participants (*F*(3, 150) = 3.11, *p* <  0.05) and the Bonferroni post hoc test showed that this difference was significant between the OSFED group and the AN group. The OSFED group also reported a significantly longer duration of illness (*F*(3, 150) = 7.58, (*p* < 0.001)), and this difference was significant compared with both the AN group and the BN group. The values of the self-report questionnaires at admission are listed in Table 2. The analyses (one-way ANOVA) indicated significantly higher scores on all measures for BN patients (*p* < 0.05 – *p* < 0.01) compared with AN patients. Overall, the level of symptom scores and BMI confirmed the severity of ED in the sample.

**Table 2:** Baseline self-report questionnaire scores for the whole sample and for the diagnostic groups

Values	Whole sample *N* = 150	AN *n* = 99	BN *n* = 32	OSFED *n* = 19	*F*-value *p*-value	Bonferroni (post hoc)
	Mean (SD)	Mean (SD)	Mean (SD)	Mean (SD)		
EDE-Q global score (*n* = 75) Range	4.3 (1.2) 0.6–5.9	4.1 (1.4) 0.6–5.7	5.0 (0.8) 2.8–5.9	4.5 (0.9) 2.6–5.8	3.41*	AN vs BN*
EDI-2 sum score (*n* = 143) Range	112.2 (40.2) 19–213	105.0 (37.6) 19–198	133.9 (44.6) 50–213	112.6 (33.7) 37–157	6.54**	AN vs BN**
EDI-2 symptom scale (*n* = 143) Range	39.7 (13.4) 8–69	36.5 (12.4) 8–66	49.1 (13.7) 23–69	40.7 (10.9) 14–51	12.09***	AN vs BN***
EDI-2 psychological scale (*n* = 143) Range	72.5 (31.6) 10–164	68.4 (30.4) 10–164	84.8 (34.4) 15–150	71.9 (27.8) 145–114	3.25*	AN vs BN*
BDI-II sum score (*n* = 139) Range	31.6 (11.1) 2–58	29.7 (10.4) 2–50	35.3 (12.2) 12–56	35.0 (10.4) 19–58	4.14*	AN vs BN*
SCL-90-R total score (*n* = 144) Range	1.7 (0.7) 0.2–3.3	1.6 (0.7) 0.2–3.2	2.0 (0.7) 0.7–3.3	1.8 (0.6) 0.6–2.6	3.79*	AN vs BN*
CIP total score (*n* = 144) Range	1.6 (0.6) 0.1–3.2	1.5 (0.6) 0.1–2.9	1.9 (0.6) 0.4–3.2	1.6 (0.05) 0.8–2.7	5.23**	AN vs BN**

*Notes:* One-way analyses of variance (ANOVA) were performed between diagnostic groups, EDE-Q *df* (2, 72), EDI-2 *df* (2, 140), BDI-II *df* (2, 136), SCL-90, and CIP *df* (2, 141. Bonferroni post hoc test; significant relationships are reported

Due to low numbers, three participants with a binge eating disorder (BED) diagnosis were included in the OSFED group

*Abbreviations: AN* anorexia nervosa, *BN* bulimia nervosa, *OSFED* other specified feeding or eating disorder, *EDE-Q* Eating Disorder Examination Questionnaire, *EDI* Eating Disorder Inventory, *BDI* Beck Depression Inventory, *SCL* Symptom Checklist, *CIP* Circumplex of Interpersonal Problems

*P* value: * *p* < .05; ** *p* < .01; *** *p* < .001

### The specialist eating disorder unit and treatment program

The specialist ED unit (Regionalt kompetansesenter for spiseforstyrrelser (RKSF)) was established in 2003 as part of the regional adult psychiatric services and primarily serves the Central Norway Regional Health Authority. Most patients are aged 18 years or older, but individuals as young as 16 years can be referred for inpatient treatment. The treatment program applied at RKSF has been described in earlier papers [23, 24].

### Measures

Self-report questionnaires were administered in accordance with routine assessment procedures, and the following were assessed: eating disorder symptoms, depression, general psychopathology, interpersonal problems, and body mass index.

#### Eating disorder symptoms

The Eating Disorder Inventory (EDI) is designed to measure symptoms, attitudes and behaviors associated with EDs [25]. Since the EDI-3 was not available in Norwegian in 2003, the EDI-2 was used in the study; a Norwegian translation became available 2015. The EDI-2 has 91 items subdivided into 11 subscales. Three subscales contribute to a symptom scale (core ED symptoms), and eight subscales to a psychological scale. The questionnaire has been validated in Nordic populations [26, 27]. The EDI-2 sum score, the symptom scale and psychological scale were used in analyses, and 143 participants at admission, 130 at discharge and 140 at follow-up completed the questionnaire. Cronbach’s alpha coefficients in the EDI-2 sum score were .95 at admission and .97 at both discharge and follow-up.

The Eating Disorder Examination Questionnaire (EDE-Q) is derived from the Eating Disorder Examination [28]. The questionnaire assesses core ED attitudes and behavior over the past 4 weeks. The EDE-Q consists of 28 items, covering 4 subscales and self-reported ED behavior [29]. The questionnaire was published in Norwegian in September 2008 and Norwegian EDE-Q norms have been established [30, 31]. In RKSF, the EDE-Q was included as an assessment tool in 2009 and therefore it was not available for the participants who had been admitted earlier. The questionnaire was completed by 75 participants at admission, 71 at discharge and 142 at follow-up. The EDE-Q global score and reports of ED behavior at follow-up were used in the definition of remission. However, due to the low number of completed questionnaires at admission and discharge, the EDE-Q was not included in analyses for the second study aim (investigation of predictors of remission). Cronbach’s alpha coefficients in the EDE-Q global score were respectively 0.94, 0.96 and 0.97 at admission, discharge and follow-up.

#### Depression

The Beck Depression Inventory (BDI-II) measures severity of depression [32]. The inventory consists of a 21-item scale. The questionnaire was completed by 139 participants at admission, 131 at discharge and 142 at follow-up; each patient’s sum score was used in the analyses. Cronbach’s alpha coefficients were respectively 0.89, 0.93 and 0.95 at admission, discharge and follow-up.

#### General psychopathology

The Symptom Checklist-90-Revised (SCL-90-R) is used to evaluate psychological problems and identify symptoms [33], and it was included in the study as a measure of general psychopathology. It has 90 items and 10 subscales. The questionnaire was completed by 144 participants at admission, 129 at discharge and 148 at follow-up; the mean total score for each patient was included in the analyses. Cronbach’s alpha coefficients were 0.97 at admission and 0.98 at both discharge and follow-up.

#### Interpersonal problems

The patients’ interpersonal problems were measured using the Circumplex of Interpersonal Problems (CIP), which has been shown as having acceptable psychometric properties [34]. The CIP is a short version of the 64-item Inventory of Interpersonal Problems (IIP64) and contains 48 items [35]. The questionnaire was completed by 144 participants at admission, 130 at discharge and 147 at follow-up; the mean total score for each participant was used in the analyses. Cronbach’s alpha coefficients in the total scores were respectively 0.93, 0.94 and 0.95 at admission, discharge and follow-up.

#### Body mass index

BMI was calculated using the formula kg/m^2^. Height was measured by the unit’s staff at admission. Participants were weighed by the staff at admission and discharge, and weight was self-reported at follow-up.

### Definition of remission

There is no consensus in the literature on how to operationalize remission in a transdiagnostic sample. However, a number of researchers have highlighted the importance of adopting consistent and shared definitions [14, 15, 17, 36]. Bardone-Cone et al. have proposed an approach that can be applied to transdiagnostic samples consisting of psychological elements, ED behavior and a physical component [15]. Based on the above-cited researchers’ recommendations and available data, we adopted the following definitions:

- Remission: EDE-Q global score within one SD of national norms (≤ 2.5) [31, 32], no binge/purge behavior in the last 4 weeks and BMI ≥ 18.5
- Partial remission: EDE-Q global score within two SDs of national norms (≤ 3.6), binge/purge behavior less than once per week and BMI ≥ 17.5
- Poor or no remission: EDE-Q global score above two SDs of national norms (> 3.6), or binge/purge behavior more than once per week or BMI < 17.5.

### Statistical analyses

In this paper descriptive data are reported both as mean values with corresponding standard deviations (SDs) for continuous variables, and as frequencies and relative frequencies for categorical variables. Differences in baseline values between diagnostic groups were investigated by one-way ANOVA with Bonferroni post hoc tests. Paired *t*-tests were used to analyze changes in questionnaire scores and BMI from admission to follow-up and from discharge to follow-up, and effect sizes were calculated (Cohen’s *d*). Chi-square tests were used to investigate both changes in ability to perform main occupation, and full-time sick leave from admission to follow-up. Logistic regression models were estimated in order to investigate predictors of outcome. Covariates in the analyses were questionnaire scores and BMI at admission, and difference scores (change from admission to discharge) in the same measures. In addition, years of illness (self-reported), age at admission, length of admission (days), and length of follow-up period (months) were included as covariates. Both univariate and multivariate models were estimated. Diagnostic groups were included as a factor in the multivariate predictor analyses. In order to simplify the multivariate model, we chose to remove factors with an estimated odds ratio (OR) that was very close to one that did not alter the ORs of the remaining covariates. Cronbach’s alpha represented the degree of internal consistency in the questionnaires. Acceptable significance levels of two-tailed analyses were set at *p* < 0.05. Analyses were performed using SPSS Statistics version 25 and STATA 14.

### Missing data

In total, 5 participants at admission, 17 at discharge and 1 at follow-up did not complete any self-report questionnaires. Some participants were missing data in single questionnaires at the different measuring points. The number of completed questionnaires at each measuring point is reported in the section “Measures” (above)*.* Eight participants did not report their weight at follow-up, and therefore their BMI could not be calculated. Complete case analyses were performed.

## Results

At follow-up, 91.3% (*n* = 137) of the participants reported that they had received outpatient specialist treatment after discharge, and 28.5% had been readmitted for inpatient treatment during the follow-up period. The participants’ reported ability to carry out their main occupation as a full-time activity (study or job) had increased significantly from 23.3% (*n* = 35) at admission to 38.1% (*n* = 61) (*X*^*2*^ (2), = 10.36, *p* < 0.01) at follow-up. The percentage of patients on full-time sick leave was significantly reduced from 36.7% (*n* = 55) at admission to 15.7% (*n* = 25) (*X*^*2*^ (2), = 15.34, *p* < 0.001) at follow-up. Three patients died after discharge and were classified as lost to follow-up, giving a mortality rate of 1.3%.

### Study aim 1—remission at follow-up

Classification of remission including the criteria of each of the three remission groups was accomplished for 142 participants, as 8 of the total 150 participants in the sample could not be classified due to missing data. At follow-up, 66.7% (*n* = 66) of AN patients had a BMI ≥ 18.5. However, if one criterion was not met, the patient was categorized in the more severe group. In total, 35.2% (*n* = 50) of the participants were classified as being in remission, 14.8% (*n* = 21) as in partial remission and 50% (*n* = 71) as having poor or no remission. The highest percentage of participants in remission was found among AN patients 38.6% (*n* = 37). The rates of remission at follow-up for the whole sample and for the diagnostic groups are presented in Table 3.

**Table 3:** Rates of remission at follow-up for the whole sample and for diagnostic groups

Remission group	Whole sample *N* = 142	AN *n* = 96	BN *n* = 29	OSFED *n* = 17
Remission EDE-Q global score ≤ 2.5, no binge/purge behavior in last 4 weeks, BMI ≥ 18.5	35.2% (*n* = 50)	38.6% (*n* = 37)	31.0% (*n* = 9)	23.5% (*n* = 4)
Partial remission EDE-Q global score ≤ 3.6, binge/purge behavior less than once per week, BMI ≥ 17.5	14.8% (*n* = 21)	13.5% (*n* = 13)	20.7% (*n* = 6)	11.8% (*n* = 2)
Poor or no remission EDE-Q global score > 3.6 or binge/purge behavior more than once per week or BMI < 17.5	50.0% (*n* = 71)	47.9% (*n* = 46)	48.3% (*n* = 14)	64.7% (*n* = 11)

*Note:* Eight participants could not be classified in remission groups due to missing data

*Abbreviations: AN* anorexia nervosa, *BN* bulimia nervosa, *OSFED* other specified feeding or eating disorder

### Study aim 2—predictors of outcome at follow-up

As a basis for prioritizing which covariates to include in the predictor analyses, the values for questionnaire scores and BMI are listed in Table 4. For the whole sample significant improvement (*p* < 0.001) was found from admission to follow-up in all mean questionnaire scores and in BMI in the AN group. Calculated effect sizes (Cohen’s *d*) ranged from moderate to high (0.46–1.76). The changes in questionnaire scores and BMI were achieved during inpatient treatment, and no further significant changes were found from discharge to follow-up.

**Table 4:** Self-report questionnaire scores and BMI in complete cases from admission to follow-up

Values	Admission	Discharge	Follow-up	Differences A–F	Effect size A–F
	Mean (SD)	Mean (SD)	Mean (SD)	*t*-value^a^; *p*-value^b^	Cohen’s *d*
EDI-2 symptom scale A–F (*n* = 133)	39.7 (13.4)	30.9 (14.8)	28.5 (17.3)	7.06***	0.67
EDI-2 psychological scale A–F (*n* = 133)	72.5 (31.5)	52.0 (32.1)	50.3 (32.5)	7.13***	0.65
BDI II sum score A–F (*n* = 133)	31.6 (11.1)	19.6 (12.9)	19.7 (14.2)	9.89***	0.92
SCL-90-R mean score A–F (*n* = 142)	1.7 (0.7)	1.1 (0.7)	1.1 (0.8)	8.46***	0.75
CIP mean score A–F (*n* = 141)	1.6 (0.6)	1.3 (0.6)	1.3 (0.7)	5.64***	0.46
BMIͨͨ AN patients^c^ A–F (*n* = 96)	15.7 (1.6)	19.6 (1.9)	19.6 (2.8)	13.62***	1.76

*Notes:*

*Abbreviations: EDE-Q* Eating Disorder Examination Questionnaire, *EDI* Eating Disorder Inventory, *BDI* Beck Depression Inventory, *SCL* Symptom Checklist, *CIP* Circumplex Interpersonal Problems

^a^Paired sample *t*-test from admission to follow-up; A – admission; F – follow-up

^b^Only significant *p*-values are reported, and *p*-value *** = *p* < .001; Cohen’s *d*: small effect size = 0.2, medium = 0.5, large = 0.8

*n* = Number of participants included in analyses

^c^Changes in body mass index (BMI) are only reported for AN patients

Among all included covariates, three were found significant predictors of remission at follow-up in the final multivariate model in the whole sample: (1) the difference in BMI from admission to discharge (OR = 1.58, *z* = 2.55, *p* < 0.05); (2) the core ED symptoms at admission measured by the EDI-2 symptom scale (OR = 0.93, *z* = − 2.60, *p* < 0.001); and (3) the difference in core ED symptoms during inpatient treatment (OR = 0.93, *z* = − 2.85, *p* < 0.01). The results indicated that having lower levels of core ED symptoms at admission, achieving higher BMI during inpatient treatment, and having a reduction in the core ED symptoms during inpatient treatment increased the probability of remission at follow-up. Compared with the final multivariate model, the univariate analyses showed more significant predictors (length of inpatient stay, and admission values of EDI-2 psychological scale, BDI-II, SCL-90, and CIP). These differences in the significance level of covariates between the models highlighted the impact of the correlation between covariates in the more complex model. The values of all covariates in the multivariate model are listed in Table 5.

**Table 5:** All predictors of remission at follow-up in the whole sample and significant values

Covariates	Logistic regression multivariate model			
	OR	95% CI	*z*-value	*p*-value
Age (years) at admission	0.92	0.76; 1.11	−0.92	0.36
Duration of illness (years)	1.04	0.85; 1.29	0.38	0.70
Follow-up period (months)	1.02	0.99; 1.04	1.20	0.23
Inpatient stay (days)	NAª	–	–	–
BMI (A)	1.16	0.99; 1.35	1.85	0.07
Diff BMI (A–D)	1.40	1.03; 1.89	2.31	< 0.05
EDI-2 symptom scale (A)	0.94	0.89; 0.99	−2.26	< 0.05
Diff EDI-2-symptom scale (A–D)	0.93	0.89; 0.99	−2.61	< 0.01
EDI-2 psychological scale (A)	NAª	–	–	–
Diff EDI-2 psychological scale (A–D)	1.01	0.99; 1.04	0.96	0.38
BDI-II sum score (A)	1.02	0.92; 1.12	0.32	0.75
Diff BDI-II (A–D)	1.04	0.96; 1.12	0.90	0.37
SCL-90-R mean score (A)	0.63	0.11; 3.63	−0.52	0.60
Diff SCL-90-R (A–D)	0.24	0.05; 1.24	−1.70	0.09
CIP mean score (A)	0.84	0.23; 3.03	−0.27	0.79
Diff CIP (A–D)	NA^b^	–	–	–

*Note:* Duration of illness was self-reported

*Abbreviations: OR* odds ratio, *AN* anorexia nervosa, *Diff* difference, *BMI* body mass index, *EDE-Q* Eating Disorder Examination Questionnaire, *EDI* Eating Disorder Inventory, *BDI* Beck Depression Inventory, *SCL* Symptom Checklist, *CIP* Circumplex Interpersonal Problems, *CI* confidence interval, *NA* not available, *A* admission, *D* discharge

ªThe odds ratio = 1 in the initial multivariate model; removed from the final model

^b^Removed from the final multivariate model due to strong correlation with CIP scores at admission that created issues of collinearity in the multivariate model

## Discussion

We investigated follow-up data in a sample of adult female patients treated at a specialized inpatient ED unit in Norway. Our results are reported for the whole (transdiagnostic) sample, as well as for three diagnostic groups (AN, BN and OSFED). At follow-up, one-third of all participants were classified as being in remission, on average 2.7 years after discharge. The AN group showed the highest proportion of remission, with 4 out of 10 participants classified as in remission. From admission to follow-up, significant changes in questionnaire scores were found in the whole sample and significant changes in BMI among AN patients, but no significant changes from discharge to follow-up. The investigation of predictors of remission at follow-up indicated that lower scores for core ED symptoms at admission, achievement of increased BMI and reduced core ED symptoms during inpatient treatment increased the probability of remission at follow-up, both in analyses performed in the whole sample and in the AN group. These findings were in accordance with our hypotheses.

Across different study samples and treatments, it has been reported that approximately half of ED patients recover and that increased remission and recovery rates result from longer follow-up periods [37]. In our adult female sample, with a mean age at admission of 21.7 (SD = 4.9) years, about half of the participants across the ED diagnoses were classified as in remission or in partial remission at follow-up. In an inpatient sample with longstanding ED and mean age at admission of 30.0 (*SD* = 7.6) years, 14% were reported as recovered and 12% partly recovered at the two-year follow-up [6]. In two studies that investigated long-term outcome (10-year and 20-year follow-up), remission was respectively 30 and 40% for AN patients and 38 and 42% for BN patients [8, 9]. In both studies, admission age was 24.9 (*SD* = 7.2) years for AN patients and 25.9 (*SD* = 7.5) years for BN patients. The participants investigated at two-year follow-up by Ro et al. were on average older than those in our study and lower remission rates were found compared with the rates for our sample [6]. Our results are in accordance with the reported long-term remission rates reported by Fichter et al. [8], and by Quadflieg & Fichter [9]. The results reported in the aforementioned studies ([6, 8, 9]) relate to severely ill ED patients who were receiving specialist inpatient treatment. However, direct comparisons of the rates of remission are challenging due to differences in the treatment programs, outcome definitions, and sample characteristics. In our study, remission was defined merely on the basis of ED-related measures and in accordance with our defined diagnostic criteria. The definition is in line with suggestions by Bardone-Cone et al. [15], and is in accordance with a study showing that the factor ‘Lack of Symptomatic Behavior’ was evaluated as the most important factor of remission/recovery from the perspective of patients, family members and clinicians [38]. However, we recognize that since recovery can be defined in different ways the differences might have had a considerable influence on the numbers of recovered patients reported for different studies [39]. Hence, there is a need to reach consensus on the definition of remission and recovery for patients with EDs to enable valid comparisons of findings across studies.

In our study, the participants in the OSFED group were on average older and reported a longer duration of their illness. A lower remission rate was found among these participants compared with participants in the AN and BN groups. However, the OSFED participants were few in number and they formed a very heterogonous group. It is important to note that the findings regarding this group are preliminary and need to be replicated in future studies.

For most patients, the admission to inpatient care was part of an extended course of treatment. As described above, there were significant improvements on all measures from admission to discharge and no change from discharge to follow-up both on measures of ED symptomatology and BMI (for patients with AN). This underlines the importance of establishing a healthy weight during inpatient care or that those who manage to reduce their underweight have a better prognosis at follow-up. However, it is important to note that in our study only 69.4% of all eligible patients participated at all three time points. Although we know that the non-participants were similar to the participants in age and BMI (AN) at admission, we have no record of their course of illness. A substantial proportion of our sample could not be classified as in remission at follow-up. However, significant symptom improvement during inpatient treatment was found for the whole sample, and the improvement was sustained at follow-up. The finding that the main symptom change was achieved during inpatient treatment supports previous findings by other researchers [6, 10, 40, 41], and may support the utility of inpatient treatment for patients with severe ED.

Many studies have investigated predictors of remission [17], but few have investigated adult inpatient samples, and the findings reported to date vary between samples and for different follow-up periods [8]. There are also differences among investigated samples (e.g., diagnosis, age and treatment program) and in chosen statistical methods. These differences must be considered in interpretation of the published results. We found an indication of increased probability of recovery in BN compared with AN. However, the BN values are associated with a high degree of uncertainty due to the low number of patients with a BN diagnosis. Other researchers have found single subscales of the EDI-2 as predictors of outcome at follow-up [8, 9, 11]. We chose to include the symptom scale of the EDI-2 as a measure of core ED symptoms (drive for thinness, bulimia, body dissatisfaction), and found it a significant predictor across diagnoses. This finding contributes important information and supports the importance of targeting the symptoms in transdiagnostic treatment programs. BMI at admission was not significant. One explanation for this finding can be related to the estimated length of inpatient stay in the specialist ED unit, which was based on the weeks required for underweight patients to reach BMI 20. In this situation, change is what matters for remission, and the admission BMI may not be as important. Other researchers have found age at admission and follow-up period are predictors of outcome [8, 9]. In our study, neither the time covariates (illness duration, length of inpatient stay, follow-up period) nor age at admission were found significant predictors of outcome at follow-up. These findings were not in accordance with our hypotheses. However, this might have been due to the sample size in our study, which was smaller than in the above-mentioned studies.

A recommended length of inpatient stay for patients with AN has not yet been agreed upon, but lower BMI at admission has been found associated with increased length of stay [42]. Decisions regarding inpatient stay are complicated and may be influenced by external factors such as health care system, economy, and availability of treatment [42–44]. In our study, the importance of weight restoration during inpatient treatment as a predictor of remission is highlighted. To enhance the rate of remission and recovery among underweight patients, the length of stay may be vital [11, 19].

### Strengths and limitations

We investigated the course of ED after inpatient treatment in a realistic clinical setting, where there were no comorbidity exclusion criteria for starting inpatient treatment. The study was strengthened by the relatively large sample size, together with the results of previous investigations of the suitability of remission definitions across diagnoses and increased knowledge of predictors of remission in transdiagnostic samples. Furthermore, due to the organization of the Norwegian health care system, with free hospitalization for everyone, the patients in our study had relatively long inpatient admissions compared with that described in most previous studies [44]. This implies that even patients with severe underweight could be in inpatients care until reaching their goal weight of BMI 20, thus maximizing the effect of inpatient care.

However, our study had some limitations, which should be noted in any interpretation of the results. All patients were recruited from the same specialist ED unit, and there was no control group available for comparisons. Due to the severity criterion for admission for the specialist inpatient treatment, the results may not be representative of the general ED population. Most of our measures were self-reported, including weight at follow-up. From previous studies, it has been shown that patients with EDs tend to overestimate their height [45], patients with AN tend to overestimate their weight and conversely patients with BN have a tendency to underestimate their weight [45, 46]. Thus, anthropometric measurements of height and weight would have been useful. However, this would have been difficult to implement due to the geographically large intake area covered by the unit. Moreover, the fact that a proportion of the eligible patients were lost to follow-up, leaving a participation rate of 69.4%, is a limitation because we cannot be certain that the non-participants were not systematically different from the participants.

The definitions of remission used in our study were based on definitions proposed by Bardone-Cone et al. [15], but with some modifications. Due to the available data, our reports have included 4 weeks without ED behavior, which is a shorter period than recommended [15]. To make comparisons between studies easier, various researchers have highlighted that it is crucial to establish definitions that are more standardized than at present [13–17]. Furthermore, our definition of remission was based on ED-related measures only. This might undermine the importance of changes in other important areas, such as patients’ general functioning, quality of life, and their own appraisal of change. In addition, adding a proxy measure of general physical health, such as the resumption of menses for underweight patients [47], could have given valuable information about the patients’ health condition. It is also necessary to consider that we had only one follow-up measure, and there was considerable variation in the length of follow-up period among participants. However, the length of the follow-up period was included in regression analyses and was not found a predictor of remission in either the univariate model or the multivariate model. Moreover, putative predictors such as purging are not included in this paper because relevant data were not available until the introduction of the EDE-Q in the unit in 2009. Furthermore, most participants received outpatient treatment following the index admission, and 28.5% received additional inpatient treatment. The latter two factors complicate the interpretation of the impact of the index hospitalization. However, as described above, length of follow-up was not a significant predictor of remission.

## Conclusions

All participants in our transdiagnostic sample had an ED requiring inpatient treatment at admission to the specialist unit for ED in Central Norway. Approximately one-third of the participants could be classified as in remission at ca. 2.5 years follow-up. However, significant changes in questionnaire scores and BMI confirmed symptom improvement during inpatient treatment and those improvements were sustained at follow-up. Increased probability of remission at follow-up was indicated by lower core ED symptoms at admission for all patients, raised BMI during admission for patients with AN, and reduced core ED symptoms during inpatient treatment for all patients. This finding contributes important information and highlights the importance of targeting these core symptoms in transdiagnostic treatment programs. To summarize, our results indicate that remission is possible for patients with EDs who need inpatient treatment, but also that many such patients have only a modest effect from the specialized treatment they receive. Because inpatient treatment is costly, the need for more personalized treatment approaches to meet needs of the most severely ill ED patients is underlined.

## Data Availability

Not applicable.

## Abbreviations

AN: Anorexia nervosa
ANOVA: Analyses of variance
BDI: Beck depression inventory
BED: Binge eating disorder
BMI: Body mass index
BN: Bulimia nervosa
CIP: Circumplex of Interpersonal Problems
DSM: Diagnostic and statistical manual **of** mental disorders
ED: Eating disorder
EDE-Q: Eating disorder examination questionnaire
EDI: Eating disorder inventory
IIP: Inventory of interpersonal problems
OR: Odds ratio
OSFED: Other specified feeding or eating disorder
SCL: Symptom checklist
SD: Standard deviation
